# Protection from reinfection in “*Candidatus* Mycoplasma turicensis”-infected cats and characterization of the immune response

**DOI:** 10.1186/1297-9716-43-82

**Published:** 2012-12-06

**Authors:** Marilisa Novacco, Felicitas S Boretti, Marco Franchini, Barbara Riond, Marina L Meli, Regina Hofmann-Lehmann

**Affiliations:** 1 Clinical Laboratory, Vetsuisse Faculty, University of Zurich, Winterthurerstrasse 260, Zurich, 8057, Switzerland; 2 Clinic for Small Animal Internal Medicine, Vetsuisse Faculty, University of Zurich, Winterthurerstrasse 260, Zurich, 8057, Switzerland; 3 Institute of Virology, Vetsuisse Faculty, University of Zurich, Winterthurerstrasse 260, Zurich, 8057, Switzerland

## Abstract

“*Candidatus* Mycoplasma turicensis” (CMt) is a hemoplasma species of felids. Recent evidence has shown that cats that overcome bacteremia may be protected from reinfection. The purposes of this study were to (1) re-inoculate ostensibly recovered cats, (2) evaluate the immune response and (3) assess CMt tissue loads. Fifteen specified pathogen-free cats were subcutaneously inoculated with CMt: 10 cats (group A) had previously undergone bacteremia and recovered, and 5 naïve cats (group B) served as controls. CMt infections were monitored by real-time PCR using blood and tissue, and the humoral immune response was assessed using DnaK ELISA. Cytokine mRNA expression levels were measured by real-time PCR, and lymphocyte subsets were detected by flow cytometry. The cats in group A were protected from reinfection (no detectable bacteremia) and showed a transient decrease in antibodies. Eosinophilia was noted in cats from group A. The cats from group B became PCR-positive and seroconverted. All of the tissues analyzed from the cats in group B but none of the tissues analyzed from the cats in group A were CMt PCR-positive. Significant changes were observed in the expression of tumor necrosis factor-α, interferon-γ, interleukin-4 and the Th2/Th1 ratio in both groups. The cats from group A occasionally showed higher numbers of CD4^+^, CD8^+^, CD4^+^CD25^+^ and CD5^+^MHCII^+^ T lymphocytes than the control cats. In conclusion, this study describes, for the first time, the occurrence of immunological protection within the same hemoplasma species. Furthermore, the immune response during CMt infections appeared to be skewed toward the Th2 type.

## Introduction

“*Candidatus* Mycoplasma turicensis” (CMt) was first isolated in a cat with hemolytic anemia [1]. During the acute phase of the infection, CMt can induce mild to moderate anemia in experimentally infected domestic cats but anemia does not always result [1]. The pathogenic potential of the different feline haemoplasma species varies and co-factors, such as immunosuppression, co-infections with other hemoplasma species or retroviral infections, may increase the severity of the disease [2]. CMt has been reported worldwide with different prevalence. In Europe the prevalence ranges from 1 to 2.3% [2]. The route of transmission is still not completely understood, but blood sucking arthropods are suspected to play a role [3-6]. Aggressive interactions between cats as well as blood transfusions have also been associated with transmission of hemotropic mycoplasma [7,8]. In experimental transmission studies, the intraperitoneal, intravenous and subcutaneous inoculation of infectious blood was successfully used to induce infection [9-11]. Experimental transmission studies have shown that CMt-infected animals develop bacteremia within 14 to 45 days post-exposure and remain PCR-positive for 10 to 21 weeks after inoculation [1,11]. Infected cats show evidence of seroconversion 10 to 47 days after exposure, and their antibody levels increase during the bacteremia [11-13]. Recently, we reported a long-term follow-up of CMt-infected cats using a newly developed *Mycoplasma haemofelis* (*Mhf*) DnaK enzyme-linked immunosorbent assay (ELISA) [12-14]. The assay has been applied to quantify anti-DnaK antibodies in cat experimentally infected with *Mhf,* “*Candidatus* Mycoplasma haemominutum” and CMt [13-16]. Interestingly, intermediate to high levels of antibodies were observed in cats that had recovered from CMt bacteremia for more than 1 year after inoculation [12]. The cats in this study were CMt-negative in their blood samples as determined by real-time TaqMan® PCR, but low bacterial copy numbers were detected in some of their tissues [12]. A preliminary experiment indicated that CMt-seropositive cats may be protected from a peak bacteremia subsequent to a repeated challenge with CMt; however, this study lacked the necessary control group [13]. The aim of the present study was to confirm and investigate this potential immunological protection during a subsequent CMt challenge under well-controlled experimental conditions. In addition, to characterize the immune response, antibody levels were assessed using *Mhf* DnaK ELISA, and cytokine mRNA profiles were measured by real-time Taqman® PCR. Furthermore, leukocyte subsets were monitored by flow cytometry.

## Materials and methods

### Animals and experimental design

Fifteen adult, age-matched, neutered, male, specified pathogen-free (SPF) cats from Liberty Research, Inc. (Waverly, New York, USA) were used for this study. The SPF status was confirmed prior to this study [11]. The cats were housed in groups in a confined university facility under ethologically and hygienically ideal conditions [17]. All experiments were approved by the veterinary office of the canton Zurich (TVB 159/2010) and were conducted in accordance with Swiss laws. Ten cats (group A) were subcutaneously infected with CMt-positive blood 26–31 months prior to this study, as previously described [11]. The cats in group A had undergone a previous experimental CMt infection and recovered from CMt bacteremia: they were PCR-negative for CMt in the peripheral blood, and 9 of the 10 cats were serologically positive for a well-recognized hemoplasma antigen, *Mhf* DnaK. Long-term follow-up of the cats in group A was conducted, as previously reported [12]. Five cats (group B) that had never been exposed to CMt prior to this study served as naïve controls for the inoculation of CMt. The cats in group B were confirmed to be PCR-negative for feline hemoplasmas and seronegative for *Mhf* DnaK. All 15 cats were inoculated with CMt by subcutaneous injection of 10 μL of blood containing 1 × 10^3^ copies of CMt diluted with 90 μL phosphate buffered saline (PBS), as previously described [11]. Ethylenediaminetetraacetic acid (EDTA)-anticoagulated blood and plasma samples were collected from all cats prior to the CMt challenge, and blood samples were collected on days 1, 2, 3 and 7 and weekly thereafter for hematology, PCR analysis and serology. The cats were monitored for 83 days post-inoculation (pi). Samples for PCR and serology were stored at −80°C within 2 h after collection. Complete hemograms were performed using a Sysmex XT-2000iV (Sysmex Corporation, Kobe, Japan) that had been previously evaluated for feline blood samples [18]. Packed cell volume (PCV) values of 33 - 45% (5 - 95% quantiles of the reference range) were considered to be within the reference range, and anemia was defined as having a PCV < 33%. For white blood cell differential analysis, microscopic blood smears were evaluated. Two blood smears were stained with a modified Wright stain using an automated staining instrument for each blood sample (Hema Tek 1000, Bayer AG, Zurich, Switzerland). Two technicians with more than 10 years of experience in veterinary hematology independently differentiated 100 cells per smear. Following the conclusion of this study, 5 cats in group A were adopted, and all other cats remained at the facility.

### Tissue sample collection

To determine whether CMt bacteria were present in the organs, tissue samples were collected under short-duration general anesthesia and analgesia by fine needle aspiration (FNA), as previously described [12]. Tissues were collected on day 30 pi from the cats in group A and on day 31 pi from the cats in group B. Tissue samples were collected from the kidney, liver and salivary glands. In addition, saliva swabs and bone marrow aspirates were collected. Bone marrow aspirates were collected from the proximal humerus under short-duration general anesthesia using a previously described protocol [11].

### Nucleic acid extraction

Total nucleic acid (TNA) was purified from 100 μL of EDTA-anticoagulated blood using the MagNaPure LC Total Nucleic Acid Isolation Kit (Roche Diagnostics, Rotkreuz, Switzerland). DNA was purified from the tissue samples using the DNA Micro Kit (Qiagen, Hombrechtikon, Switzerland). DNA from the bone marrow samples was purified using the DNA Blood and Tissue kit (Qiagen) according to the manufacturer's instructions. For each batch of extractions, negative controls consisting of 200 μL of phosphate-buffered saline were concurrently prepared to monitor for cross-contamination.

### Quantitative TaqMan® real-time PCR assays

All blood and tissue samples were tested in triplicate by real-time TaqMan® PCR for the presence and bacterial load of CMt, as previously described [1]. In addition, a TaqMan® real-time PCR reaction amplifying the pseudogene glyceraldehyde-3-phosphate dehydrogenase (GAPDH) [19] was included for all tissue samples to confirm the presence of amplifiable DNA and the absence of significant PCR inhibitors [12]. Only samples with more than 5 000 copies of GAPDH/PCR reaction were considered acceptable, and the DNA extraction was repeated for samples that produced insufficient copies. CMt tissue loads were normalized by dividing the CMt copy number by the GAPDH copy number [12,20]. In each real-time PCR reaction, a positive control and 2 negative controls consisting of nucleic acid-free water were included.

### Serology

Antibodies against the *Mhf* DnaK protein were detected and quantified in naïve and serologically positive cats after CMt inoculation using an ELISA assay, as described [14]. The serum was diluted by 1:100 in wells, and 50 ng of recombinant protein per well was used. Wells containing antigen without serum served as blanks, and wells containing pre-infection serum samples served as negative controls. The ELISA signal-to-noise ratio was calculated by dividing the post-infection absorbance values by the pre-infection absorbance values for each individual cat, as previously described [14]. An ELISA signal-to-noise ratio of ≥ 1.5 was considered positive.

### RNA isolation and cDNA synthesis

Blood samples for cytokine transcriptional analysis were collected on days 1, 3, 7, 14, 21, 28 and 35 pi from all cats in both groups. One hundred microliters of EDTA-anticoagulated blood was collected and mixed immediately with 300 μL of mRNA lysis buffer (mRNA Isolation Kit I, Roche Diagnostics, Rotkreuz, Switzerland). The samples were stored at −80°C within 1 h of collection. mRNA was purified using the mRNA Isolation Kit I and a MagNA Pure LC instrument (Roche Diagnostics) according to the manufacturer's instructions. For all extractions, negative controls consisting of 100 μL of phosphate buffered saline were prepared alongside each batch to monitor for cross-contamination. mRNA was eluted in 25 μL of elution buffer and was stored at −80°C until further use. First-strand cDNA was synthesized using the High Capacity cDNA Reverse Transcription Kit (Applied Biosystems, Rotkreuz, Switzerland) according to the manufacturer's instructions. For each mRNA sample, cDNA was synthesized in duplicate and then pooled. The cDNA samples were stored at −20°C until used in the PCR measurements.

### Real-time TaqMan® PCR for quantification of cytokine expression

Real-time TaqMan® PCR was used for the relative quantification of feline interferon-γ (IFN-γ), interleukin (IL) 4, 6, 10 and 12 and tumor necrosis factor-α (TNF-α). The PCR assays used in this study have been described elsewhere [19,21,22]. V-abl Abelson murine leukemia viral oncogene homolog (ABL) and zeta polypeptide (YWHAZ) transcription levels served as reference genes for normalization, and these genes were quantified by real-time PCR, as previously described [23]. PCR assays were performed using a Rotor-Gene 6000 real-time rotary analyzer (Corbett, Mortlake, Australia). Negative and positive controls were included in each PCR run. The calculation of mRNA expression levels was performed using GeNorm version 3.5 [24].

### Preparation and labeling of lymphocytes and flow cytometry

EDTA-anticoagulated blood samples were collected from the cats in groups A and B before CMt inoculation and on days 1, 2, 3 and 7 and weekly thereafter until day 56 pi. The blood samples were processed within 1 h after collection. Tubes were gently rotated end-over-end for 5 min at room temperature before cell preparation. Then, the blood samples were divided into 100 μL aliquots and placed in 5 mL Falcon polystyrene tubes (BD Biosciences, Allschwil, Switzerland). The aliquots were incubated at 4°C in the dark for 30 min with one of the following antibody combinations: 1) a fluoresceinisothiocyanate (FITC)-conjugated mouse anti-feline CD5 antibody (f43, Southern Biotech, Allschwil, Switzerland) recognizing T lymphocytes [25] and an unconjugated mouse anti-feline MHCII antibody (H34A, VMRD); 2) an R-phycoerythrin (RPE)-conjugated mouse anti-feline CD4 antibody (Vpg34, AbD serotec, Düsseldorf, Germany) and a fluoresceinisothiocyanate (FITC)-conjugated mouse anti-feline CD25 antibody (kindly provided by MB Tompkins, North Carolina State University, USA); 3) an unconjugated mouse anti-feline CD8 antibody (FT2, Southern Biotech) and a peridinin chlorophyll-a protein (PerCP)-conjugated rat anti-mouse CD45R/B220 antibody (RA3-6B2, BD Bioscience). The antibodies were diluted as recommended by the manufacturer, and an aliquot of blood from each cat was used unstained as an isotype-matched control antibody. After incubation with the antibody, the samples were treated with 2 mL of Tris-NH_4_Cl (pH 7.2) for 7–8 min to selectively lyse the red blood cells. The samples were centrifuged at 600 × *g* for 10 min to pellet the leukocytes. The leukocyte pellet was incubated with secondary antibodies or with PBS containing 2% fetal calf serum (Gibco Life Technologies, Basel, Switzerland) at room temperature for 20 min. Allophycocyanin (APC)-conjugated mouse IgG1 anti-CD8 antibody (BD Pharmingen) and an RPE-goat anti-mouse IgG2b MHCII antibody (Southern Biotech) were used for staining leukocytes. All antibodies used in the current study have been previously used in feline species [26,27]. After antibody incubation, the cells were washed with PBS containing 2% fetal calf serum and were centrifuged at 600 × *g* for 10 min to pellet the leukocytes. The cells were resuspended in 250 μL of PBS containing 2% fetal calf serum and were immediately subjected to flow cytometry. The cells were enumerated and differentiated using the BD FACSCalibur™ platform (Becton Dickinson, Allschwil, Switzerland) and the CellQuestPro™ software. Gates representing lymphocytes were set based on forward versus side scatter, and 10 000 events were acquired for each sample. Each lymphocyte subset was calculated by multiplying the absolute lymphocyte number (determined by the complete blood cell count) by the subset percentage (expressed as the percentage of lymphocytes determined by flow analysis), as previously described [28]. The data were analyzed using FlowJo software (Tree Star, Olten, Switzerland).

### Statistics

Statistical analyses were performed using GraphPad Prism for Windows version 3.0 (GraphPad software, San Diego, CA, USA). The Mann–Whitney U-test (*P*_MWU_) was used to compare parameters between groups, and the Wilcoxon signed rank test (*P*_Wilcoxon_) was applied for paired analyses. Friedman’s test (*P*_Friedman_), followed by Dunn’s post-test (*P*_Dunns_), was used to analyze the parameters over time. Correlation analyses were performed using Spearman’s rank order correlation coefficient test (r_*s*_). *P*-values < 0.05 were considered to be significant.

## Results

### Clinical and laboratory parameters

None of the cats in groups A or B developed clinical signs indicative of a hemoplasma infection (e.g., pallor, lethargy, fever or weight loss). Six out of the 10 cats from group A showed intermittent PCV values below the reference range (PCV < 33%, 5% quantile) starting at day 1 pi, and the lowest PCV value observed in group A was 27%. All five cats in group B showed PCV values < 33% during the observation period; however, 2 cats from group B had PCV values of 30% and 32% on day 0. The lowest observed PCV value in group B was 22%. The cats from group B had significantly lower PCV values than the cats from group A after the onset of bacteremia (days 35, 70 and 83 pi, *P*_MWU_ < 0.05).

Five out of the 10 cats from group A showed leukocytosis (> 12 800/μL, 95% quantile) on occasion throughout the observation period. Additionally, 2 cats from group A (cats FHX4 and FHX5) demonstrated increased leukocyte counts during the entire study (up to 21 400/μL), including on day 0. The cats from group A showed higher leukocyte counts when compared with the cats from group B on days 0, 1, 7, 14, 21, 28, 35, 42, 63 and 70 (*P*_MWU_ < 0.05).

Lymphocyte counts were significantly higher in the cats from group A when compared with the cats from group B on day 28 pi (*P*_MWU_ = 0.0047). Overall, 3 cats from group A demonstrated lymphocyte counts above the reference range (6 000/μL, 95% quantile), and 2 of them (cats FHX4 and FHX5) showed consistently higher lymphocyte counts throughout the observation period, including day 0, which is indicative of lymphocytosis (up to 10 070/μL).

Differences between groups A and B were also noted in neutrophil and monocyte counts at different time points of the experiment, but the majority of the values remained within the reference range (46 - 678/μL and 2 315–10 011/μL, 5 - 95% quantiles, for monocytes and neutrophils, respectively). The cats from group A showed higher neutrophil counts when compared with the cats from group B on days 0, 1, 7, 21, 35, 42, 63 and 70 (*P*_MWU_ < 0.05). Higher monocytes counts were found in cats from group B on days 35 and 42 pi compared to the cats from group A.

In contrast, eosinophilia (values > 600/μL, 95% quantile) was detected in the cats from group A, and 7 out of the 10 cats from group A demonstrated eosinophilia throughout the entire study, including day 0 (up to 2 290/μL). A significant difference in eosinophil numbers was detected between groups A and B from day 28 pi until the end of the observation period (*P*_MWU_ < 0.05); this trend was also present, albeit not quite significant, on days 7, 14 and 21 pi (*P*_MWU_ = 0.0553).

### Protection from CMt challenge in ostensibly recovered cats

**Figure 1:**
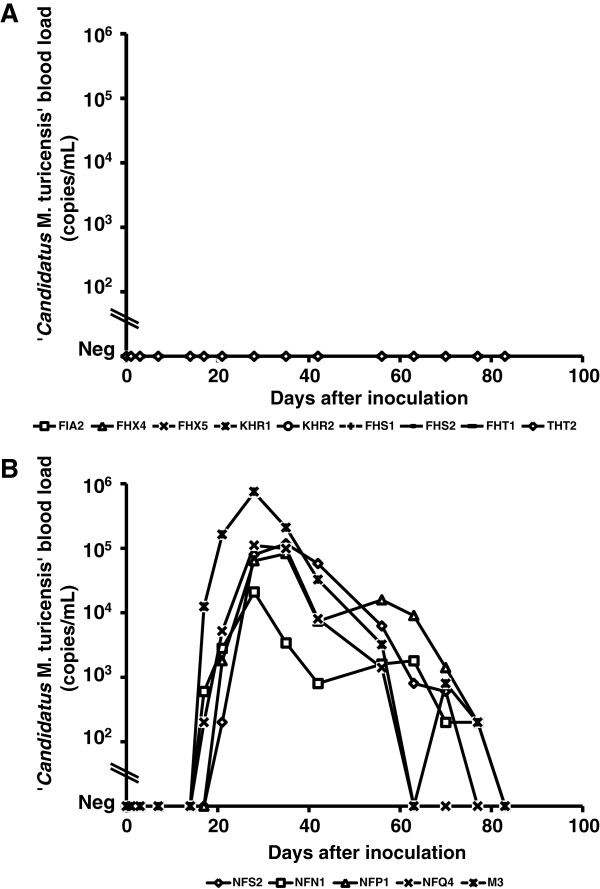
**CMt blood loads in fifteen SPF cats after CMt inoculation.** Cats in groups A (**A**) and B (**B**) were inoculated subcutaneously at day 0 and were monitored for 83 days. CMt blood loads (y-axes) are expressed as the log copy numbers per mL of blood. The cats in group **A** showed no detectable bacteremia upon CMt inoculation, whereas the cats in group **B** became CMt PCR-positive after an average of 19 days (range: 17–21 days).

The cats from group A remained PCR-negative in blood throughout the entire study (Figure 1A). The cats from group B became PCR-positive after an average of 19 days (range: 17–21 days pi) with peak blood loads ranging from 10^4^ - 10^5^ copies/mL of blood between 28–35 days pi (Figure 1B). In all tissue samples, a sufficient amount of amplifiable DNA and the absence of PCR inhibitors were confirmed using GAPDH TaqMan® real-time PCR. All five cats from group B showed detectable CMt tissue loads (Table 1), whereas no CMt DNA was detected in any of the tissue samples analyzed for the cats in group A (Table 1).

**Table 1:** **CMt tissue loads (copies/10**^**6**^**cells) determined by real-time Taqman® PCR in tissue samples collected at days 30 and 31 pi for cats in groups A and B**

**Group**	**Cat**	**Kidney**	**Liver**	**Salivary gland**	**Bone marrow**	**Saliva swab**
**A**	FIA1	-	-	-	-	-
	FIA2	-	-	-	-	-
	KHR1	-	-	-	-	-
	KHR2	-	-	-	-	-
	FHS1	-	-	-	-	-
	FHS2	-	-	-	-	-
	FHT1	-	-	-	-	-
	FHT2	-	-	-	-	-
	FHX4	-	-	-	-	-
	FHX5	-	-	-	-	-
**B**	M3	70 684	169	25 140	680 319	61
	NFN1	376	2 555	298 501	6 066	26
	NFP1	576	387	13 871	244 306	402
	NFQ4	12 801	10 568	6 039	2 080	495
	NFS2	15 138	40 981	27 389	738 300	0

- = CMt tissue loads were below the detection limit of PCR [7].

### Humoral immune response

**Figure 2:**
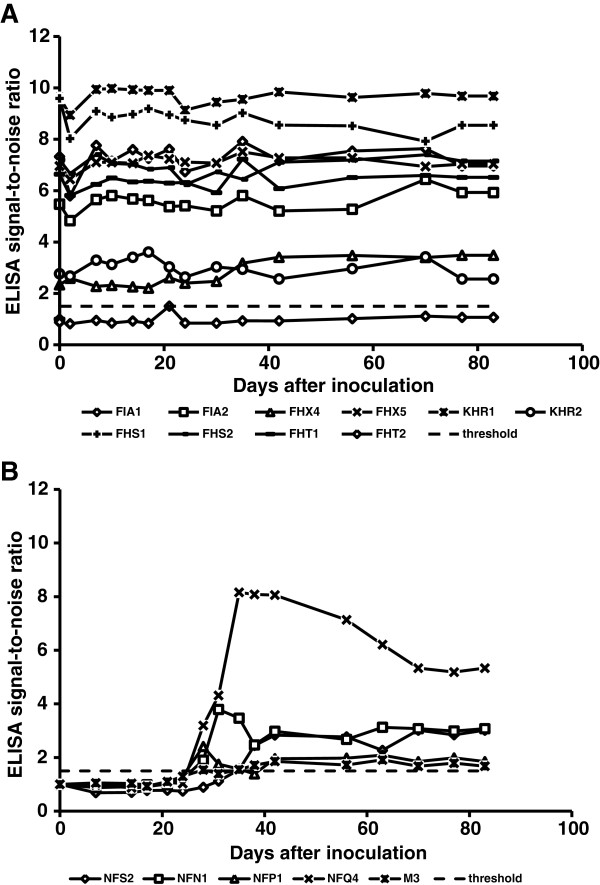
**Humoral immune response to *Mhf *DnaK after CMt exposure.** Cats in groups A (**A**) and B (**B**) were inoculated subcutaneously with CMt on day 0 and were monitored for 83 days. Antibody levels are shown as signal-to-noise ratios (y-axes). The cats in group **A** showed a significant transient decrease in antibody levels 1 day after inoculation, whereas seroconversion was detected in the cats from group **B** between 28 and 35 days pi. A signal-to-noise ratio of 1.5 is indicated by a dashed line, which represents the defined threshold for seropositivity.

At the beginning of this study, 9 out of the 10 cats from group A were seropositive (Figure 2A). One cat (cat FHT2, group A) showed a signal-to-noise ratio ≤ 1.5 by ELISA and was therefore considered seronegative (Figure 2A). However, this cat also showed protection from CMt reinfection (Figure 1A). Remarkably, a significant transient decrease in antibodies was observed in the cats from group A on day 1 pi (*P*_Wilcoxon_ = 0.0098), which was followed by a significant increase in antibody levels by day 2 pi (*P*_Wilcoxon_ = 0.0059) (Figure 2A). No significant differences in antibody levels were detected over time in the cats from group A. The cats from group B were seronegative prior to CMt inoculation and became seropositive between 28 and 35 days pi (Figure 2B). The cats from group B remained seropositive until the end of the observation period.

### Evaluation of the cytokine response

**Figure 3:**
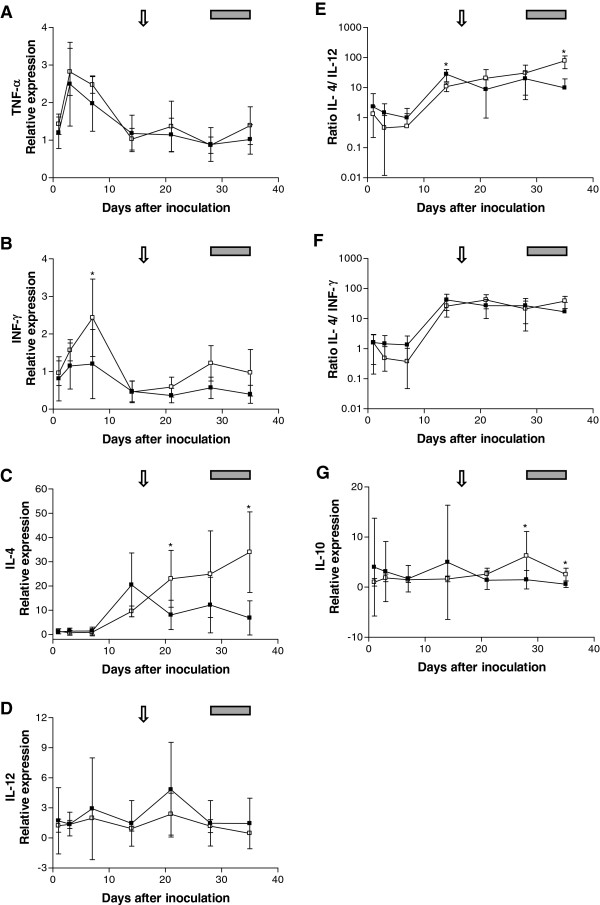
**Relative expression levels of TNF-**α **(A), IFN-**γ **(B), IL-4 (C), IL-12 (D) and IL-10 (G) after CMt inoculation on day 0 in cats from group A (black squares) and group B (open squares).** The Th2/Th1 ratio is expressed as the IL-4/IL-12 ratio (**E**) and the IL-4/IFN-γ ratio (**F**). Cytokine mRNA expression levels were measured by real-time Taqman® PCR and were normalized to the expression levels of two housekeeping genes (ABL and YWHAZ) at each time point. The values shown represent the mean and standard deviation for each group of cats. The arrow indicates the onset of bacteremia and the gray box indicates the peak bacteremia for the cats in group **B**. Relative expression levels were tested for statistical differences between groups by the Mann–Whitney U-test (asterisks indicate statistically significant differences; *P* < 0.05).

CMt inoculation induced an approximately 2.5-fold upregulation of TNF-α within the first week in the cats from groups A and B (*P*_Wilcoxon_ = 0.0020 and *P*_Wilcoxon_ = 0.0625 for groups A and B, respectively; Figure 3A). However, the TNF-α levels returned to basal values (level before CMt inoculation) within 14 days pi in both groups. No significant differences in TNF-α levels were detected between groups A and B.

In the cats from group A, IFN-γ expression levels were statistically significantly higher at day 3 compared to day 35 pi (*P*_Wilcoxon_ = 0.0039, Figure 3B). The cats in group B showed a nearly 2.5-fold upregulation of IFN-γ expression from day 1 to day 7 pi (Figure 3B), which resulted in significantly higher IFN-γ levels in the cats from group B compared to the cats from group A on day 7 (*P*_MWU_ = 0.0400). Thereafter, the IFN-γ levels decreased significantly (*P*_Wilcoxon_ = 0.01) to slightly below basal levels by day 14 and day 21 pi in group B.

IL-4 expression levels changed significantly over time in both groups (*P*_Friedman_ < 0.001). The cats from group A showed increased IL-4 levels on day 14 (approximately 20-fold upregulation), but this trend was only transient, with significantly lower levels in comparison to group B on days 21 (Figure 3C; *P*_MWU_ = 0.0177) and 35 (Figure 3C; *P*_MWU_ = 0.0101). The IL-4 expression levels in group A were statistically significantly higher on days 14, 21, 28 and 35 pi when compared with the IL-4 levels from day 1 (*P*_Wilcoxon_ < 0.05) and remained increased by approximately 10-fold until the end of the observation period. The cats from group B showed an increase in IL-4 expression levels after day 7 and the IL-4 levels in group B cats continuously increased until day 35 pi. IL-4 levels in group B cats were statistically significantly higher at day 35 pi compared to days 1, 3 and 7 pi (*P*_Dunns_ < 0.05; Figure 3C), peaking at an approximately 30-fold higher level of IL-4 when compared with the basal levels (Figure 3C). In group B, the IL-4 levels correlated significantly with the CMt bacterial blood load (r_*s*_ = 0.82; *P* < 0.0001).

No significant differences were found in IL-12 levels after CMt inoculation between the two groups throughout the observation period (Figure 3D). We further calculated the ratio of IL-4 to IL-12 expression levels as a surrogate marker for the T helper 2 (Th2)/T helper 1 (Th1) ratio. Both groups of cats showed an increase in their Th2/Th1 ratio between days 7 and 14 pi (Figure 3E; *P*_Wilcoxon_ = 0.0078 and *P*_Wilcoxon_ = 0.0625 in groups A and B, respectively). The cats from group A showed a significantly higher Th2/Th1 ratio on day 14 than the cats from group B (Figure 3E; *P*_MWU_ = 0.0031), whereas the cats from group B showed a higher Th2/Th1 ratio than the cats from group A on day 35 pi (Figure 3E; *P*_MWU_ = 0.0025). When Th2/Th1 ratios were calculated using IL-4 and IFN-γ as the surrogate markers, similar results were obtained: an increase in the Th2/Th1 ratio in both groups was observed between day 7 and day 14 pi (Figure 3F; *P*_Wilcoxon_ = 0.0078 and *P*_Wilcoxon_ = 0.0625 in groups A and B, respectively). In addition, prior to the observed increase between days 7 and 14 pi, the Th2/Th1 ratio decreased from day 1 to day 7 in group B (Figure 3F; *P*_Wilcoxon_ = 0.0625), while no differences were noted in group A.

IL-10 expression levels changed significantly over time in cats from group A (Figure 3G; *P*_Friedman_ = 0.0138). In the cats from group A, IL-10 expression levels were statistically significantly higher at day 35 compared to days 1 and 3 pi (*P*_Dunns_ < 0.05, Figure 3G). No significant differences in IL-10 levels over time were detected in group B cats. IL-10 expression levels in the cats from group A were significantly lower compared to the cats from group B on days 28 and 35 (*P*_MWU_ = 0.0047). The IL-6 expression levels were below the detection limit of real-time PCR in 41 out of 105 samples tested (data not shown). Therefore, the IL-6 levels were excluded from the statistical analysis.

### Leukocyte subsets after CMt inoculation

**Figure 4:**
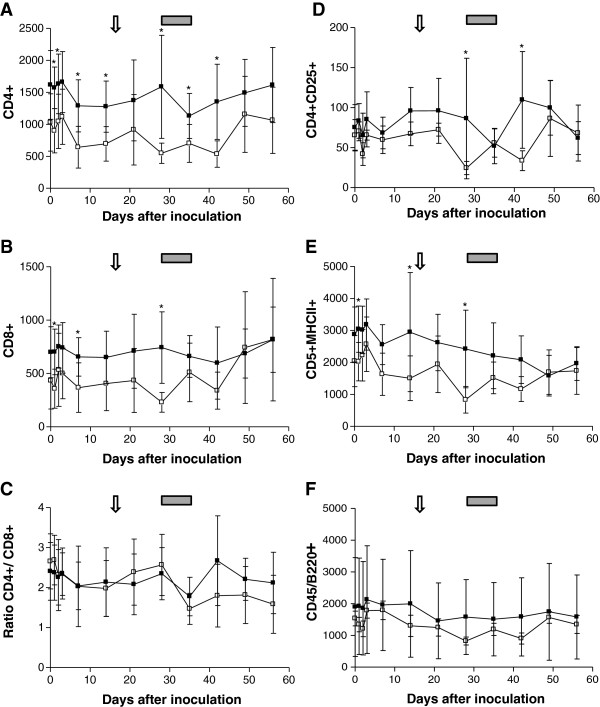
**Dynamics of the CD4**^**+**^**(A), CD8**^**+**^**(B), CD4**^**+**^**/CD8**^**+**^**(C), CD4**^**+**^**CD25**^**+**^**(D), CD5**^**+**^**MHCII**^**+**^**(E) and B220**^**+**^**(F) cell populations after CMt inoculation on day 0 in cats from group A (black squares) and group B (open squares).** The values shown represent the mean and standard deviation for each group of cats. The arrow indicates the onset of bacteremia and the gray box represents the peak bacteremia for the cats in group **B**. Activation markers were tested for statistical differences between groups by the Mann–Whitney U-test (asterisks indicate statistically significant differences; *P* < 0.05).

No significant differences in any of the tested cell subsets were detected between groups A and B on day 0. After CMt inoculation, significant changes in the CD4^+^ T cell counts were detected in groups A and B (*P*_Friedman_ < 0.001). In particular, the cats from group A showed a statistically significant decrease in CD4^+^ T cells from day 2 to day 14 pi (Figure 4A; *P*_Dunns_ < 0.05). This decrease was followed by an increase in CD4^+^ cells from day 35 to day 56 (Figure 4A; *P*_Dunns_ < 0.05). The cats from group B showed a statistically significant decrease in CD4^+^ cells from day 2 to day 42 (Figure 4A; *P*_Dunns_ < 0.05) followed by a significant increase from day 42 to day 49 pi (Figure 4A; *P*_Dunns_ < 0.05). The cats from group A showed higher CD4^+^ T cell counts when compared with the cats from group B on days 1, 2, 7, 14, 28, 35 and 42 (Figure 4A; *P*_MWU_ < 0.04).

No statistically significant changes over time were noted in the CD8^+^ T cell populations during the observation period in group A or B. However, the cats from group A showed significantly higher numbers of CD8^+^ T cells when compared with cats from group B on days 1, 7 and 28 pi (Figure 4B; *P*_MWU_ < 0.05). In addition, we calculated the CD4^+^/CD8^+^ ratio (Figure 4C) and found that it changed significantly during the observation period in cats from groups A and B (*P*_Friedman_ < 0.001). Both groups showed a decrease in the CD4^+^/CD8^+^ ratio between day 28 and day 35 pi; however, this decrease was only significant in group B (Figure 4C; *P*_Dunns_ < 0.05). We also observed a subsequent increase in the CD4^+^/CD8^+^ ratio between day 35 and day 42, which was significant in group A (*P*_Dunns_ < 0.001; Figure 4C). No statistical differences were noted between groups A and B.

Significant changes in the CD4^+^CD25^+^ T cell counts were detected during the observation period in both groups (*P*_Friedman_ < 0.001). In particular, two significant decreases in the CD4^+^CD25^+^ cell population were detected in the cats from group A on day 35 and day 56 pi (Figure 4D; *P*_Dunns_ < 0.05). The first decrease in CD4^+^CD25^+^ T cells was followed by a significant increase between day 35 and day 42 pi (Figure 4D; *P*_Dunns_ < 0.01). The cats from group B showed a statistically significant decrease in the CD4^+^CD25^+^ counts on day 28 pi (Figure 4D; *P*_Dunns_ < 0.05), which was followed by a significant increase on day 49 pi when compared with day 28 pi (Figure 4D; *P*_Dunns_ < 0.05). Higher CD4^+^CD25^+^ T cell counts were also observed on days 28 and 42 pi in the cats from group A relative to the cats from group B (Figure 4D; *P*_MWU_ < 0.006).

Significant changes in CD5^+^MHCII^+^ T cells were found in groups A and B over time (*P*_Friedman_ < 0.001). The cats from group A demonstrated a statistically significant lower CD5^+^MHCII^+^ T cell levels at days 42, 49 and 56, respectively, compared to day 3 pi (Figure 4E; *P*_Dunns_ < 0.05). A transient decrease in CD5^+^MHCII^+^ T cell levels was also noted in the cats from group B on day 28 and day 42 relative to day 2 pi (Figure 4E). Significantly higher CD5^+^MHCII^+^ T cell levels were noted in the cats from group A relative to the cats from group B on days 1, 14 and 28 pi (Figure 4E; *P*_MWU_ < 0.04).

A significant decrease in B220^+^ cell levels was detected in the cats from group A on days 35, 42 and 56 relative to day 3 pi (Figure 4F; *P*_Dunns_ < 0.05). No significant changes in B220^+^ cell levels were noted in the cats from group B, and no statistically significant changes were noted in B220^+^ cell levels between groups A and B.

## Discussion

The present study describes, for the first time, the immunological protection from reinfection in cats that overcome CMt bacteremia under well-controlled experimental conditions. Cats that had ostensibly overcome the acute phase of CMt infection without antibiotic treatment were re-inoculated with CMt and showed protection from a second bacteremia. In contrast, the naïve control cats became PCR-positive in blood and tissue samples after CMt inoculation. The mechanisms involved in the protection of these cats have not been completely elucidated by our study, but our evidence suggests a significant role of the humoral and cellular immune responses in this protection.

We evaluated the humoral immune response to CMt to detect possible changes in antibody levels after CMt inoculation. The cats that had overcome a previous bacteremia showed intermediate to high levels of antibodies before CMt challenge. A boost in CMt-specific antibodies could have been expected due to the repeated exposure; however, the majority of cats from group A showed a significant transient decrease in antibodies upon CMt reinfection. A similar decrease in antibody was recently described in a preliminary study investigating repeated CMt exposure [13]. In that study, the authors speculated that the decrease in serum antibodies may be related to the binding of specific free antibodies to the inoculated antigens [13]. Remarkably, one cat in the group of CMt recovered cats was seronegative based on our definition (ELISA signal-to-noise ratio ≤ 1.5); however, this cat maintained protection against reinfection. This observation suggests that specific antibodies against *Mhf* DnaK are not the sole protective mechanism against reinfection in these cats. *Mhf* DnaK is a hemoplasma antigen that is largely used in experimental studies to detect seroconversion after hemoplasma infection [12,13,15,16]. Other antigens have been recognized in blood samples from *Mhf*-infected cats [29,30], but their immunological potential is unknown. The entire feline hemoplasma genome has been recently sequenced [31,32]; thus, information regarding additional antigens may soon become available. In serological assays, the combination of different antigens should be considered for a better understanding of the kinetics of the humoral immune response during hemoplasma infection.

In this study, we demonstrated that CMt was present in all analyzed tissues of the naïve control cats upon acute infection, as high tissue loads were found, particularly in the bone marrow. In contrast, the ostensibly recovered cats tested PCR-negative in all tissues analyzed, including the bone marrow. Thus, it appears that the cats were not only protected from bacteremia but were also sufficiently protected from recurrent tissue sequestration to prevent detection based on repeated real-time PCR measurements in the analyzed tissues.

To further characterize the immune response after CMt exposure in naïve control cats and recovered cats, we quantified different cytokine expression levels. Immediately after CMt inoculation, all cats showed a significant increase in TNF-α secretion. This cytokine is the principal mediator of the acute inflammatory response [33]. TNF-α is produced by activated mononuclear phagocytes, and the function of TNF-α is to stimulate the recruitment of neutrophils and monocytes to sites of infection [33]. We suspect that the subcutaneous inoculation of CMt stimulated the release of TNF-α, and it was indeed detected in acutely infected as well as in re-exposed cats.

The Th1/Th2 paradigm has been previously described in cats [34]. Thus, we were able to select cytokines specific for each type of T helper response for the cats in this study. Th1 cells mainly mediate immune responses against intracellular pathogens, whereas Th2 cells are mainly involved in host defense responses against extracellular pathogens and atopic diseases [35]. IL-4 stimulates the expansion of B cells and Th2-associated cells, inhibiting the proliferation of Th1 cells [36]. On the other hand, IFN-γ is secreted by Th1 cells, inducing antimicrobial activity in macrophages [37]. The cats acutely infected with CMt (naïve control cats) showed an early transient increase in IFN-γ expression (Th1 cytokine) that peaked at day 7 after CMt inoculation. In contrast, the cats that had recovered from previous CMt bacteremia did not show an early increase in IFN-γ expression but rather showed a pronounced increase in IL-4 expression (Th2 cytokine) at day 14 pi and prior to the time point corresponding to bacteremia in acutely infected cats. Thus, in the CMt recovered cats, protection from reinfection was associated with an early and pronounced Th2 response, whereas the acutely infected cats responded to CMt challenge with an initial Th1 response and a delayed Th2 response. A switch from a Th1 to a Th2 response in acutely infected cats was observed based on the evolution of the IL-4/IFN-γ ratios. An initial decrease in the ratio of IL-4/IFN-γ was followed by an increase, which was in turn followed by bacteremia. In contrast, the CMt recovered cats did not display skewed Th1/Th2 ratios based on either the IL-4/IFN-γ ratio or the IL-4/IL-12 ratio. Only after the onset of bacteremia were IL-4 levels higher in the acutely infected cats when compared with the CMt recovered cats, and the IL-4 levels were correlated with the bacterial load. Thus, an early Th2 response prior to the onset of bacteremia appears to be beneficial to the protection from CMt reinfection, while a delayed Th2 response seemed to be generated in response to the underlying bacteremia.

Interestingly and in agreement with the trend in Th2 responses, we detected a constant eosinophilia during CMt infection, which was particularly evident in the CMt recovered cats. Eosinophilia is a hallmark of a predominance of the Th2 response [38,39]. The recruitment of eosinophils, although usually associated with parasitic and allergic inflammation, can also be associated with certain bacterial infections [40]. Eosinophilia has been previously reported during feline hemoplasma infections but was not considered clinically relevant [41,42]. In addition, a previous study reported an association between eosinophilia and latent *Haemobartonella canis* infection [43]. In the context of observations by other researchers, our results may suggest that CMt infection leads to an upregulation of Th2 cell populations, which in turn stimulates the recruitment of eosinophils.

Different markers were used to analyze the lymphocyte subsets by flow cytometry after CMt inoculation. The cats that had recovered from previous bacteremia showed higher CD4^+^ T cell levels when compared with acutely infected cats at several time points after the CMt inoculation. As mentioned earlier in this discussion, our results suggest that the Th2 differentiation of CD4^+^ T cells occurs during CMt infection to efficiently combat CMt. The Th2 response is characterized by B cell activation and proliferation [37]. Thus, the immune system of CMt recovered cats appears to immediately recognize CMt antigens, and this results in rapid activation of the appropriate T helper cells. T helper cells appear to assist CMt recovered cats to fight subsequent CMt infections and prevent bacteremia by activating secondary mechanisms (e.g., antibody production). Additionally, the activation of CD4^+^ T cells was most likely responsible for the recruitment of eosinophils [44].

CD4^+^CD25^+^ T cells represent a subpopulation of CD4^+^ T cells commonly known as regulatory T cells [45]. This subset of cells suppresses the proliferation and cytokine secretion of other T cell populations and suppresses the activation of self-reacting CD4^+^ and CD8^+^ T cells [46]. Additionally, regulatory T cells may influence the functional immunity of cats during microbial infection to suppress the immune response to pathogens [47]. For this reason, CD4^+^CD25^+^ T cells were monitored during our study to understand their role during infection. CD4^+^CD25^+^ T cell levels were higher in the CMt recovered cats when compared with the acutely infected cats following the onset of bacteremia. The downregulation of the immune response induced by regulatory T cells may have promoted the persistence of the CMt infection and may have been instrumental in the establishment of the CMt carrier state. The number of regulatory T cells decreased at peak bacteremia in the acutely infected cats, which may be related to the role of these cells in regulating the immune response. With a decreased number of regulatory T cells, the acutely infected cats showed downregulation of the suppressive function of these cells, which in turn was most likely beneficial for fighting the CMt infection.

Some T cell activation marker levels were higher in the cats that had overcome previous bacteremia than in the acutely infected cats at various time points during the observation period. This could be related to the increased lymphocyte counts detected in the CMt recovered cats, as the lymphocytosis was probably due to chronic CMt antigenic stimulation. A similar phenomenon was identified during a cross-protection study in feline hemoplasmas (unpublished observations, Baumann J, Novacco M, Hofmann-Lehmann R), whereby cats chronically infected with CMt showed significant antigenic stimulation, which led to an increase in γ-globulin (unpublished observations, Baumann J, Novacco M, Hofmann-Lehmann R).

In conclusion, our data indicate that cats that overcome CMt bacteremia possess a persistent immune response skewed towards the Th2 type, which is associated with protection from reinfection with CMt. Further studies are required to better understand the immunological mechanisms involved in this protection. Characterization of the protective immune response against hemoplasmas is an initial step toward the development of effective vaccines against these types of infections.

## Competing interests

The authors declare that they have no competing interests.

## Authors' contributions

MN participated in the design of the study, carried out the *in vivo* experiments and drafted the manuscript. FB participated in the design of the study, helped with the *in vivo* experiments and revised the manuscript. MF helped with the development of the flow cytometry assay. BR was responsible for the SPF cats and revised the manuscript. MM supported the laboratory work and revised the manuscript. RH-L participated in the design and coordination of the study, helped with the data analysis and edited the manuscript. All authors read and approved the final manuscript.
